# Unintentional forgetting is beyond cognitive control

**DOI:** 10.1186/s41235-019-0180-5

**Published:** 2019-07-16

**Authors:** Ashleigh M. Maxcey, Bernadette Dezso, Emma Megla, Ashton Schneider

**Affiliations:** 1 Department of Psychology, Vanderbilt University, Nashville, TN USA; 2 Department of Psychology, The Ohio State University, 1835 Neil Ave, Columbus, OH USA

## Abstract

Intentional forgetting refers to the attempt to marshal top-down control to purposefully forget, and has been demonstrated in the laboratory using directed forgetting paradigms. Here, we asked whether the mechanisms of top-down control can run in the opposite direction to prevent the forgetting of information; that is, can we actively resist unintentional forgetting? Recognition-induced forgetting is an unintentional forgetting effect in which accessing one memory leads to the forgetting of related memories. Just as intentional forgetting possesses intuitively appealing control over eliminating undesirable memories, resisting unintentional forgetting over desired memories would improve cognitive performance. We showed subjects a 9-min video to teach them about the recognition-induced forgetting paradigm and how recognition of certain objects unintentionally leads to forgetting of semantically related objects. After testing their comprehension of the video, we conducted a typical recognition-induced forgetting experiment and challenged the subjects to resist this form of unintentional forgetting. Despite their knowledge of the forgetting effect, and the challenge to subjects to resist the forgetting induced by the paradigm, recognition-induced forgetting persisted. These results show that knowledge of this unintentional forgetting phenomenon and the challenge to resist forgetting do not eliminate it, suggesting that it is cognitively impenetrable.

**Electronic supplementary material:**

The online version of this article (10.1186/s41235-019-0180-5) contains supplementary material, which is available to authorized users.

## Significance

Control over memory is critical to our daily lives. The ability to intentionally forget, as in expunging an unpleasant memory, has been demonstrated in the laboratory. Here we ask whether the same control can be exerted over unintentional forgetting. To this end, we employed a forgetting paradigm that unintentionally causes forgetting of information when a semantically related target is retrieved from memory. We trained subjects on this forgetting effect and instructed them to resist forgetting. We found that neither briefly trained, nor expert subjects who were offered a reward, could resist forgetting. These results suggest that, unlike the control we appear to have over intentional forgetting, unintentional forgetting is beyond our control. Evidence that awareness does not bolster immunity to unintentional forgetting calls for caution under a plethora of real-world cognitive tasks in which one of several semantically related memories is targeted, such as eyewitness testimony and education.

## Introduction

Intentional forgetting refers to the attempt to marshal top-down control to purposefully forget and has been demonstrated in the laboratory using directed forgetting (MacLeod, 2012; Muther, 1965) and think/no-think paradigms (Anderson & Green, 2001). These laboratory tasks demonstrate appealing control over forgetting unwanted memories, such as an unpleasant encounter or an unwanted telephone call. Here we asked whether the mechanisms of top-down control can run in the opposite direction to prevent the forgetting of desirable memories, such as when studying for an examination or a vacation; that is, can we actively resist unintentional forgetting?

Here we use unintentional forgetting in reference to the incidental negative consequence of accessing a target memory, the forgetting of related memories (Anderson, Bjork, & Bjork, 1994; Maxcey & Woodman, 2014). Recognition-induced forgetting is a robust unintentional forgetting effect whereby practice in recognizing an item in memory leads to the forgetting of related items (Maxcey, 2016; Maxcey & Bostic, 2015; Maxcey, Bostic, & Maldonado, 2016; Maxcey, Glenn, & Stansberry, 2017; Rugo, Tamler, Woodman, & Maxcey, 2017).^1^ Although this unintentional forgetting may operate in real-world scenarios such as identifying suspects in a lineup or studying for an examination, the ability to exert executive control over this forgetting phenomenon has never been tested.

Despite arguments that forgetting is adaptive (Bekinschtein, Weisstaub, Gallo, Renner, & Anderson, 2018), one might argue that if it were possible to eliminate forgetting under circumstances in which we desired to remember, we would have already eliminated forgetting. Indeed, there is reason to believe that subjects cannot resist unintentional forgetting. First, the spreading activation nature of recognition-induced forgetting, in that the forgotten information is semantically related to a targeted memory, suggests forgetting may be automatic (Logan, 1988) and thus cognitively impenetrable. Second, manipulations to eliminate memory errors in another semantic-spreading task, the Deese–Roediger–McDermott paradigm, have been unsuccessful (McDermott, 1996). In the Deese–Roediger–McDermott task, subjects are presented with a list of semantically related words to remember (e.g., cookie, sugar). In a later memory test, subjects misreport semantically related lures (e.g., sweet) as having been on the list. Efforts to abolish this effect through repeating learned items are unsuccessful in eliminating this effect.

Conversely, there is reason to believe that subjects can resist unintentional forgetting. First, the ability to exert executive control over memory, as in directed forgetting tasks and their predecessors (Bjork, 1972; Bjork, Laberge, & Legrand, 1968; Brown, 1954; Epstein, 1972; MacLeod, 2012; Muther, 1965), suggests that some forgetting is under volitional control. Second, executive control appears to eliminate a related forgetting phenomenon, that of retrieval-induced forgetting (Imai, Kim, Sasaki, & Watanabe, 2014). Third, explanations of retrieval-induced forgetting have posited a role of executive control (Anderson, 2003), suggesting it may be possible to wield control over this effect. Fourth, the ability to exert top-down control over memory encoding has been frequently demonstrated (Adcock, Thangavel, Whitfield-Gabrieli, Knutson, & Gabrieli, 2006; Gruber & Otten, 2010; Gruber, Watrous, Ekstrom, Ranganath, & Otten, 2013) and is reflected in the neural correlates of memory encoding (Sunby, Woodman, & Fukuda, 2019), suggesting that resisting recognition-induced forgetting may be possible.

Here we asked whether subjects were able to resist recognition-induced forgetting following instructions to do so. In Experiment 1, subjects were taught about recognition-induced forgetting and challenged to resist forgetting before completing the paradigm. In Experiment 2, subjects were naive to recognition-induced forgetting to provide a baseline measure of susceptibility to this forgetting effect. In Experiment 3, recognition-induced forgetting experts were challenged to resist forgetting. A unique aspect of our experimental design is that we were able to compare susceptibility to this unintentional forgetting effect between informed subjects (Experiment 1), naive subjects (Experiment 2), and expert subjects (Experiment 3).

## General methods

**Fig. 1:**
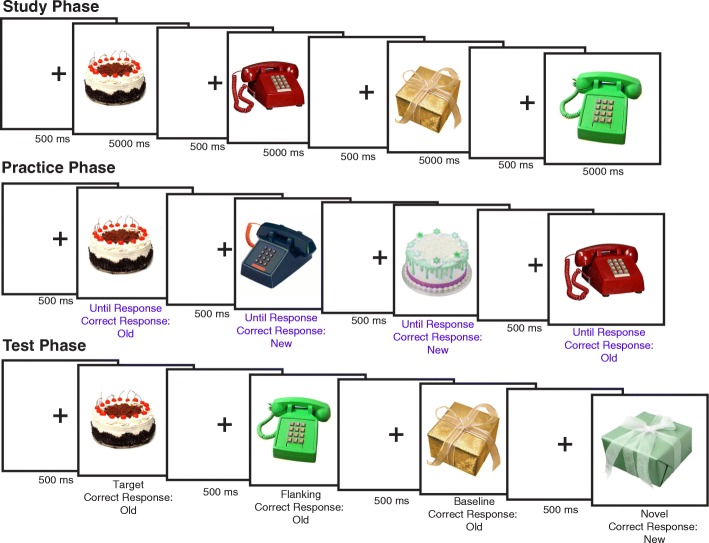
The general methods for all three experiments. In the study phase, subjects fixated a central fixation point for 500 ms, followed by the presentation of the stimuli for 5000 ms, until all stimuli were presented. The subjects were instructed to remember each item for a later memory test. The study phase was followed by a 5-min visual distractor task. In the practice phase, half of the items from half of the categories were again presented along with an equal number of novel items. Subjects engaged in recognition practice by completing an old–-new recognition judgment task in response to each item. Each old item was practiced on two practice trials. The practice lures were items drawn from the same categories as the practice items. The second phase was followed by another 5-min visual distractor task. The test phase employed the same old–new recognition judgment task as the second phase, but included 36 old stimuli from the study phase (12 baseline, 12 practiced, and 12 related), as well as an equal number of novel test lures from the same categories. Hit rates from this test phase are presented in Figs. 2, 3 and 4

The experiment began with the study phase (Fig. 1). Subjects were presented with 72 objects for 5 s each and instructed to remember each object for a later memory test. The objects were six objects drawn from 12 basic-level object categories.^2^

Next, in the recognition practice phase, subjects completed an old–new recognition judgment task testing memory for a subset of the objects from the study phase. Specifically, half of the objects from half of the categories (3 objects × 6 basic-level categories = 18 objects total) from the study phase were presented twice during the practice phase (36 old trials). For a 50/50 old–new correct response distribution, 36 new objects were included in the practice phase. The specific objects practiced were counterbalanced across subjects. A 5-min delay interval during which the subjects completed a visual distractor task followed both the study and practice phases.

In the test phase, the subject’s task was the same as the practice phase, but the total object set differed. Subjects were presented with 36 old objects and 36 new objects from corresponding object categories. Old objects in the test phase included three object types. Practiced objects were shown in the study phase and practiced in the practice phase. When subjects encountered a practiced object in the test phase, they were seeing it for the fourth time. Related objects were from the study phase that were not practiced. However, they are categorically related to practiced objects (e.g., telephones were practiced but not this specific telephone). Baseline objects were also in the study phase but not the practice phase, just like related objects. The difference between related and baseline objects is that baseline objects were from categories that were not practiced (e.g., presents were not practiced). Memory for baseline and related objects is critical to the signature recognition-induced forgetting effect. Recognition-induced forgetting is when related objects are remembered at reliably worse rates than baseline objects (Maxcey, 2016; Maxcey & Bostic, 2015; Maxcey & Woodman, 2014; Maxcey et al., 2016, 2017; Rugo et al., 2017). The 36 old objects were evenly drawn from the three object types (i.e., practiced, related, and baseline) and the 36 new objects were drawn from corresponding basic-level object categories (e.g., an equal number of telephones).

## Experiment 1

In Experiment 1, subjects watched a video describing how the recognition of certain objects unintentionally leads to forgetting of semantically related objects in the recognition-induced forgetting paradigm. Subjects were challenged to resist this forgetting before completing a recognition-induced forgetting experiment.

### Methods

#### Participants

A pilot experiment run to determine the necessary sample size (Faul, Erdfelder, Lang, & Buchner, 2007) had a d_z_ = 0.76. If we wanted to have 95% power to detect an effect equal to this with a two-tailed t-test, we would require 25 subjects. A second power analysis was run over data from Experiment 1 of our original recognition-induced forgetting study (Maxcey & Woodman, 2014) resulting in d_z_ = 1.376. If we wanted to have 99% power to detect an effect equal to this with a two-tailed *t* test, we would require 12 subjects. We ran 52 subjects knowing that we may lose subjects based on insufficient effort leading to chance performance.

Subjects were 54 Ohio State University undergraduates (mean age 19.09 years, 34 female, 20 male) who completed the experiment in exchange for course credit. The critical manipulation in this experiment relied on effortful participation, indicating that subjects whose memory for baseline objects at test were at or below chance should be excluded from analysis resulting in the loss of 16 subjects. The analysis presented here is comprised of the 38 remaining subjects (mean age 19.11 years, 24 female, 14 male). Subjects reported normal color vision and normal or corrected-to-normal visual acuity. Informed consent was obtained prior to the beginning of the experiment. All procedures were approved by the Institutional Review Board.

#### Stimuli and apparatus

Stimuli were presented on a white background on a flat-screen monitor using e-prime software (Schneider, Eschman, & Zuccolotto, 2012). The stimulus set consisted of pictures drawn from 12 basic-level object categories with 15 exemplars from each category. The specific categories practiced were counterbalanced across subjects. Subjects reported whether the pictures were old or new during the recognition practice and test phases using a Chronos response box. Subjects were seated approximately 80 cm from the monitor in a dimly lit room. Stimuli subtended approximately 4.6° of visual angle.

#### Procedure

The procedure followed the general methods described above with the following exceptions. Before beginning the experiment, subjects watched a 9-min video^3^ describing the forgetting typically induced in the recognition-induced forgetting paradigm and challenging them to resist forgetting. Then subjects completed a quiz (Additional file 1) to confirm they comprehended the video before starting the experiment. Following the experiment, subjects completed a survey (Additional file 2) to inquire about strategy and effort.

#### Data analysis

The primary dependent variable for recognition memory is hit rate across the three main object types: practiced, related, and baseline. To provide converging evidence for hit rate analyses, in footnotes beneath the critical comparisons we report the discrimination measure (*Pr*) and the associated bias measure (*Br*)^4^ (Feenan & Snodgrass, 1990). All preplanned *t* tests with equal sample size are accompanied by scaled JZS Bayes factor (Rouder, Speckman, Sun, Morey, & Iverson, 2009). Significant *t* tests are accompanied by Cohen’s *d* measure of effect size. Individual subject data are discussed in Additional file 3.

### Results

**Fig. 2:**
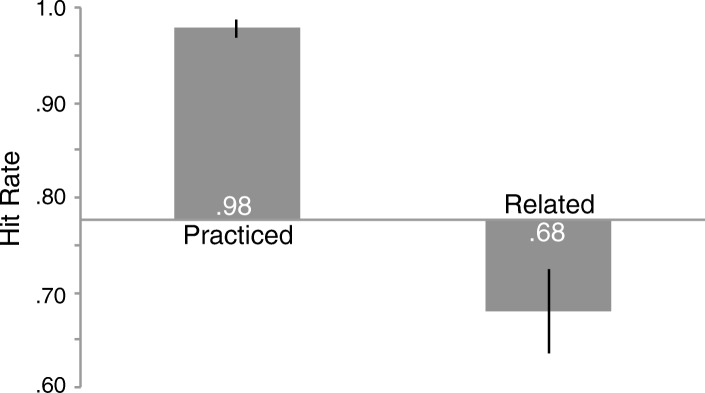
Hit rate for old objects in the test phase by object type from Experiment 1. The *x* axis represents memory for baseline objects and error bars represent 95% confidence intervals as described by Cousineau (2005) with Morey’s correction applied (Morey, 2008)

A repeated-measures analysis of variance (ANOVA) comparing the means for object type (*F* (2,74) = 58.667, *p* < .001, η_p_^2^ = .613) indicated a reliable difference (Fig. 2; see Additional file 3 for individual subject data). Memory for related objects (.68) was worse than memory for baseline objects (.78, *t* (37) = 3.421, *p* = .002, *d* = 0.58, scaled JZS_ALT_^5^ = 21.19) and memory for practiced objects (.98) was better than baseline (.78, *t* (37) = 8.313, *p* < .001, *d* = 2.14, scaled JZS_ALT_ = 20,198,134).^6^ Despite learning about recognition-induced forgetting and instructions to resist forgetting, subjects were susceptible to forgetting. Importantly, forgetting was not induced by a build-up of interference from the presentation of foils across the test phase because recognition performance in the second half of the test phase (.84) was not reliably worse than the first half (.85, *t* (37) = 0.819, *p* = .418, scaled JZS_NULL_ = 4.19).

**Fig. 3:**
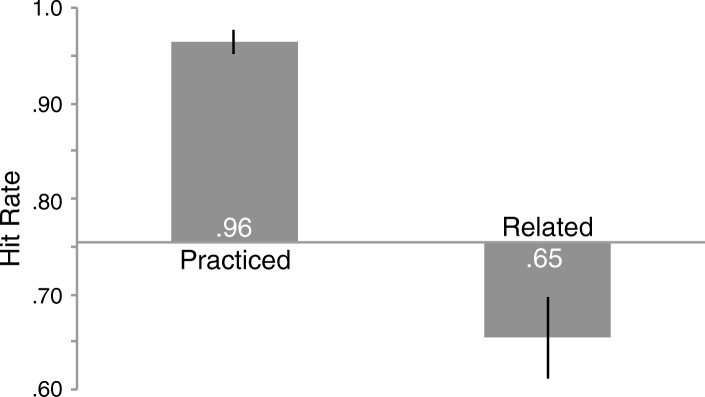
Hit rate for old objects in the test phase by object type from Experiment 2. The *x* axis represents memory for baseline objects and error bars represent 95% confidence intervals as described by Cousineau (2005) with Morey’s correction applied (Morey, 2008)

### Discussion

Despite subject’s knowledge of recognition-induced forgetting, and the challenge to resist forgetting, recognition-induced forgetting persisted. These results show that knowledge of this unintentional forgetting phenomenon and the challenge to resist forgetting do not eliminate it, suggesting that it is cognitively impenetrable.

## Experiment 2

In Experiment 2 we replicated the methods of Experiment 1 but without the instructional video to provide a baseline of the proportion of subjects that demonstrate forgetting in a typical recognition-induced forgetting experiment.

### Methods

#### Participants

Subjects were 54 Ohio State University undergraduates (mean age 19.23 years, 24 female, 30 male) who completed the experiment for course credit. To allow a comparison between experiments, the same exclusion criterion for chance memory performance was applied resulting in the loss of 15 subjects. The data reported here are from the remaining 39 subjects (mean age 19.13, 20 female, 19 male).

#### Procedure

The procedure was identical to Experiment 1 with the following exceptions. The video and quiz were not administered before beginning the experiment. A slightly different survey was administered at the end of the experiment (Additional file 4).

### Results

A repeated-measures ANOVA comparing the means across object types (*F* (2,76) = 71.740, *p* < .001, η_p_^2^ = .654) indicated a reliable difference (Fig. 3; see Additional file 3 for individual subject data). Memory for related objects (.65) was worse than memory for baseline objects (.75, *t* (38) = 3.597, *p* = .001, *d* = 0.69, scaled JZS_ALT_ = 33.31) and memory for practiced objects (.96) was better than baseline (.75, *t* (38) = 10.211, *p* < .001, *d* = 2.54, scaled JZS_ALT_ = 4,457,051,777).^7^ As in Experiment 1, forgetting was not induced by interference because recognition performance in the second half of the test phase (.80) was not reliably worse than the first half (.81, *t* (38) = 0.095, *p* = .925, scaled JZS_NULL_ = 5.77).

### Discussion

Replicating Experiment 1 and other studies on recognition-induced forgetting, naive subjects were susceptible to forgetting. Taken together, Experiments 1 and 2 show that knowledge of the recognition-induced forgetting effect did not reliably decrease susceptibility to forgetting, suggesting this forgetting effect is robust and cognitively impenetrable.

## Experiment 3

Although the post-video quiz in Experiment 1 indicated subjects understood recognition-induced forgetting, it is possible that training was insufficient to enable resistance to forgetting. Expertise in recognition-induced forgetting may eliminate susceptibility to the effect. To determine whether subjects with a thorough understanding of recognition-induced forgetting were immune to forgetting, we replicated Experiment 2 with undergraduate research assistants working on recognition-induced forgetting projects in our laboratory who were instructed to resist forgetting. These subjects served as experts who would have a better understanding of the effect than subjects in Experiment 1.

### Methods

#### Participants

Subjects were 11 Ohio State University undergraduates (mean age 20.8, 8 female, 3 male) who worked in our laboratory^8^ and completed the experiment in exchange for the possibility of a pizza reward (see Procedure).

#### Procedure

The procedure is identical to Experiment 2 with the following exceptions. Subjects were members of our laboratory who were experts on recognition-induced forgetting. Subjects were instructed to resist recognition-induced forgetting. Subjects were informed that if they successfully resisted forgetting, they would win a pizza reward.

### Results

**Fig. 4:**
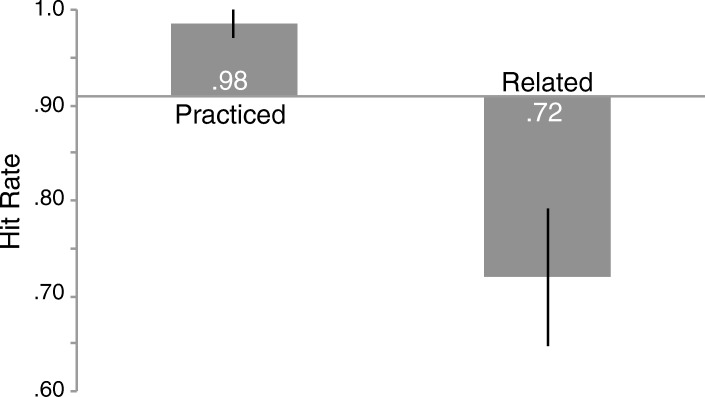
Hit rate for old objects in the test phase by object type from Experiment 3. The *x* axis represents memory for baseline objects and error bars represent 95% confidence intervals as described by Cousineau (2005) with Morey’s correction applied (Morey, 2008)

A repeated-measures ANOVA comparing the means across object type (*F* (2,20) = 18.327, *p* < .001, η_p_^2^ = .647) indicated a reliable difference (Fig. 4; see Additional file 3 for individual subject data). Memory for related objects (.72) was worse than memory for baseline objects (.91, *t* (10) = 4.202, *p* = .002, *d* = 1.36, scaled JZS_ALT_ = 25), demonstrating recognition-induced forgetting with experts. Memory for practiced objects (.98) was not significantly better than baseline (.91, *t* (10) = 1.910, *p* = .085, *d* = 1.02, scaled JZS_ALT_ = 1.2),^9^ likely due to extremely high memory performance, indicative of effort to resist forgetting. Replicating Experiments 1 and 2, forgetting was not induced by interference because recognition performance in the second half of the test phase (.90) was not reliably worse than the first half (.91, *t* (10) = 0.382, *p* = .711, scaled JZS_NULL_ = 3.16).

### Discussion

Despite extremely high baseline performance (.91) indicating a high degree of effort, expertise in recognition-induced forgetting did not eliminate the effect.

## General discussion

Here we parametrically manipulated subject’s knowledge of recognition-induced forgetting, from naive subjects (Experiment 2) to subjects who received video training on recognition-induced forgetting (Experiment 1) and expert subjects (Experiment 3). We found that only a minority of subjects was immune to recognition-induced forgetting in each group. However, overall, all three groups of subjects were susceptible to the forgetting effect.

The inability to exert top-down control over recognition-induced forgetting is surprising for several reasons. First, numerous studies demonstrate the ability to exert control over memory encoding, by upregulating or improving memory for the targeted items (Adcock et al., 2006; Gruber & Otten, 2010; Gruber et al., 2013) and downregulating or forgetting targeted memories (Anderson & Hanslmayr, 2014; Gruber et al., 2013; MacLeod, 1998, 1999). Second, control over memory is even reflected in the neural correlates of memory encoding (Sunby et al., 2019). Third, demand characteristics would suggest numerous strategies for overcoming this forgetting effect, such as purposefully performing incorrectly for the practiced or baseline items to lower overall memory performance (but see Sunby et al., 2019 for data regarding the ability to voluntarily downregulate memory). Fourth, informing the subjects of the goals and methodology of many paradigms in cognitive psychology would presumably eliminate their effects (as in Simons, 2010). For example, giving subjects advance warning that there will be a surprise memory test for items they are told to forget in directed forgetting studies would presumably ameliorate forgetting. Warning subjects that the types of questions they are asked are meant to modify memory in classic false memory tasks would likely reduce or eliminate the rate of false memories. These examples of popular memory tasks that presumably rely largely on subjects’ naivety illustrate the surprising and empirically useful persistence of recognition-induced forgetting in the present study.

This is the first experiment to test cognitive control over recognition-induced forgetting. We found that recognition-induced forgetting is cognitively impenetrable. These results are at odds with evidence that people can improve memory when offered a reward (Adcock et al., 2006; Gruber & Otten, 2010; Gruber et al., 2013; Imai et al., 2014) and when simply cued to try harder when encoding particular pictures (Sunby et al., 2019). In fact, improved memory for pictures preceded by a priority pre-cue is accompanied by electrophysiological markers of attentional resources (i.e., prestimulus occipital alpha power suppression and visually evoked N1; Sunby et al., 2019), demonstrating that it is possible to successfully exert cognitive control over memory encoding. The inability to resist forgetting here, despite the ability to exert control over information processing in general (Gaspelin, Leonard, & Luck, 2015, 2017; Hickey, Di Lollo, & McDonald, 2009; Noonan et al., 2016; Noonan, Crittenden, Jensen, & Stokes, 2018; Posner, 1980; Sawaki & Luck, 2011), indicate that models of forgetting need not involve executive control (Anderson, 2003; Kim, Lewis-Peacock, Norman, & Turk-Browne, 2014) but rather automaticity likely plays a role (Logan, 1988) in weakening associated memories (Raaijmakers, 2018). These results also suggest that the robustness of this effect may increase its empirical utility. Outside the laboratory, knowledge of memory phenomenon, such as recognition-induced forgetting, may not improve cognitive functioning and performance in a wide range of real-world scenarios, from the law to education.

## Additional files

Additional file 1: Experiment 1 post-video quiz. (DOCX 599 kb)

Additional file 2: Experiment 1 post-experiment survey. (DOCX 17 kb)

Additional file 3: Individual subject data. (DOCX 13 kb)

Additional file 4: Experiment 2 post-experiment survey. (DOCX 13 kb)

## Data Availability

The datasets analyzed during the current study are available from the corresponding author on reasonable request.

## Footnotes

1 The recognition-induced forgetting paradigm, by definition, presents additional novel pictures in the old–new recognition judgment task. Our laboratory has demonstrated that forgetting is not parametrically manipulated by set size, suggesting that forgetting is not due to retroactive interference but is indeed the result of recognition (Maxcey, 2016).

2 Basic-level object categories included telephone, cake, present. Find the full stimulus set at https://osf.io/j47hz/?view_only=c7e130d1209d4683b1618d44f01a2c5d.

3
https://www.youtube.com/watch?v=pRhce229Ets&feature=youtu.be

4 *Br* values greater than 0.5 are indicative of a liberal bias while values lower than 0.5 indicate a conservative bias. When calculating *Br*, *Pr* values of 1 were changed to 0.99.

5 JZS Bayes Factor provides a way of quantifying support for either the null or alternative hypothesis (Rouder et al., 2009). For example, here the alternative hypothesis is 38 times more likely to be true than the null. JZS_ALT_ is a value in favor of the alternative and JZS_NULL_ is a value in favor of the null hypothesis.

6 Recognition-induced forgetting replicates when using *Pr*, with baseline *Pr* (0.66) significantly higher than related *Pr* (0.56) (*t* (37) = 3.411, *p* = .002, *d* = 0.47, scaled JZS_ALT_ = 20.68), and conservative biases for both baseline (*Br* = 0.40) and related (*Br* = 0.32).

7 Recognition-induced forgetting replicates when using *Pr*, with baseline *Pr* (0.61) significantly higher than related *Pr* (0.50) (*t* (38) = 3.597, *p* = .001, *d* = 0.60, scaled JZS_ALT_ = 33.31), and liberal biases for both baseline (*Br* = 3.67) and related (*Br* = 2.50).

8 Two of the authors, BD and EM, were subjects in this experiment prior to collaborating on this project and were thus not invested in any particular outcome. We acknowledge that Experiment 3 may be underpowered as the sample size was determined by the number of research assistants in the laboratory available to serve as experts. However, rather than driving the main purpose or conclusions of the present study, Experiment 3 is an attractive enhancement of the central argument that knowledge of recognition-induced forgetting does not ameliorate its effect.

9 Recognition-induced forgetting replicates when using Pr, with baseline *Pr* (0.85) significantly higher than related *Pr* (0.66) (*t* (10) = 4.224, *p* = .002, *d* = 1.28, scaled JZS_ALT_ = 25). The bias for baseline was liberal (*Br* = 0.54) and the bias for related was conservative (*Br* = 0.23).

## References

[CR1] Adcock RA, Thangavel A, Whitfield-Gabrieli S, Knutson B, Gabrieli JD (2006). Reward-motivated learning: Mesolimbic activation precedes memory formation. Neuron.

[CR2] Anderson MC (2003). Rethinking interference theory: Executive control and the mechanisms of forgetting. Journal of Memory and Language.

[CR3] Anderson MC, Bjork RA, Bjork EL (1994). Remembering can cause forgetting: Retrieval dynamics in long-term memory. Journal of Experimental Psychology: Learning, Memory, and Cognition.

[CR4] Anderson MC, Green C (2001). Suppressing unwanted memories by executive control. Nature.

[CR5] Anderson MC, Hanslmayr S (2014). Neural mechanisms of motivated forgetting. Trends in Cognitive Sciences.

[CR6] Bekinschtein P, Weisstaub NV, Gallo F, Renner M, Anderson MC (2018). A retrieval-specific mechanism of adaptive forgetting in the mammalian brain. Nature Communications.

[CR7] Bjork RA (1972). Theoretical implications of directed forgetting. Coding processes in human memory.

[CR8] Bjork RA, Laberge D, Legrand R (1968). The modification of short-term memory through instructions to forget. Psychonomic Science.

[CR9] Brown J (1954). The nature of set-to-learn and of intra-material interference in immediate memory. Quarterly Journal of Experimental Psychology.

[CR10] Cousineau D (2005). Confidence intervals in within-subject designs: A simpler solution to Loftus and Masson’s method. Tutorial in Quantitative Methods for Psychology.

[CR11] Epstein, W. (1972). Mechanisms of directed forgetting. In *Psychology of learning and motivation* (Vol. 6, pp. 147-191). Academic Press.

[CR12] Faul F, Erdfelder E, Lang A-G, Buchner A (2007). G*Power 3: A flexible statistical power analysis for the social, behavioral, and biomedical sciences. Behavior Research Methods.

[CR13] Feenan K, Snodgrass JG (1990). The effect of context on discrimination and bias in recognition memory for pictures and words. Memory & Cognition.

[CR14] Gaspelin N, Leonard CJ, Luck SJ (2015). Direct evidence for active suppression of salient-but-irrelevant sensory inputs. Psychological Science.

[CR15] Gaspelin N, Leonard CJ, Luck SJ (2017). Suppression of overt attentional capture by salient-but-irrelevant color singletons. Attention, Perception, & Psychophysics.

[CR16] Gruber MJ, Otten LJ (2010). Voluntary control over prestimulus activity related to encoding. Journal of Neuroscience.

[CR17] Gruber MJ, Watrous AJ, Ekstrom AD, Ranganath C, Otten LJ (2013). Expected reward modulates encoding-related theta activity before an event. Neuroimage.

[CR18] Hickey C, Di Lollo V, McDonald JJ (2009). Electrophysiological indices of target and distractor processing in visual search. Journal of Cognitive Neuroscience.

[CR19] Imai H, Kim D, Sasaki Y, Watanabe T (2014). Reward eliminates retrieval-induced forgetting. Proceedings of the National Academy of Sciences.

[CR20] Kim G, Lewis-Peacock JA, Norman KA, Turk-Browne NB (2014). Pruning of memories by context-based prediction error. Proceedings of the National Academy of Sciences.

[CR21] Logan GD (1988). Toward an instance theory of automatization. Psychological Review.

[CR22] MacLeod CM, Golding JM, MacLeod CM (1998). Directed forgetting. Intentional forgetting: Interdisciplinary approaches.

[CR23] MacLeod CM (1999). The item and list methods of directed forgetting: Test differences and the role of demand characteristics. Psychonomic Bulletin & Review.

[CR24] MacLeod, C. M. (2012). Directed forgetting. *Encyclopedia of the Sciences of Learning*, 993-995.

[CR25] Maxcey AM (2016). Recognition-induced forgetting is not due to category-based set size. Attention, Perception, & Psychophysics.

[CR26] Maxcey AM, Bostic J (2015). Activating learned exemplars in children impairs memory for related exemplars in visual long-term memory. Visual Cognition.

[CR27] Maxcey Ashleigh M., Bostic Jessica, Maldonado Ted (2016). Recognition Practice Results in a Generalizable Skill in Older Adults: Decreased Intrusion Errors to Novel Objects Belonging to Practiced Categories. Applied Cognitive Psychology.

[CR28] Maxcey Ashleigh M., Glenn Hannah, Stansberry Elisabeth (2017). Recognition-induced forgetting does not occur for temporally grouped objects unless they are semantically related. Psychonomic Bulletin & Review.

[CR29] Maxcey AM, Woodman GF (2014). Forgetting induced by recognition of visual images. Visual Cognition.

[CR30] McDermott KB (1996). The persistence of false memories in list recall. Journal of Memory and Language.

[CR31] Morey RD (2008). Confidence intervals from normalized data: A correction to Cousineau (2005). Tutorial in Quantitative Methods for Psychology.

[CR32] Muther WS (1965). Erasure or partitioning in short-term memory. Psychonomic Science.

[CR33] Noonan MP, Adamian N, Pike A, Printzlau F, Crittenden BM, Stokes MG (2016). Distinct mechanisms for distractor suppression and target facilitation. Journal of Neuroscience.

[CR34] Noonan MP, Crittenden BM, Jensen O, Stokes MG (2018). Selective inhibition of distracting input. Behavioural Brain Research.

[CR35] Posner MI (1980). Orienting of attention. Quarterly Journal of Experimental Psychology.

[CR36] Raaijmakers JGW, Wixted JT, Phelps EA, Davachi L (2018). Inhibition in memory. Stevens’ handbook of experimental psychology and cognitive neuroscience (4th ed., vol. 1, Learning and Memory).

[CR37] Rouder JN, Speckman PL, Sun D, Morey RD, Iverson G (2009). Bayesian t-tests for accepting and rejecting the null hypothesis. Psychonomic Bulletin & Review.

[CR38] Rugo KF, Tamler KN, Woodman GF, Maxcey AM (2017). Recognition-induced forgetting of faces in visual long-term memory. Attention, Perception, & Psychophysics.

[CR39] Sawaki R, Luck SJ (2011). Active suppression of distractors that match the contents of visual working memory. Visual Cognition.

[CR40] Schneider W, Eschman A, Zuccolotto A (2012). E-Prime reference guide.

[CR41] Simons DJ (2010). Monkeying around with the gorillas in our midst: Familiarity with an inattentional-blindness task does not improve the detection of unexpected events. i-Perception.

[CR42] Sunby C, Woodman GF, Fukuda K (2019). Electrophysiological and behavioral evidence for attentional up-regulation, but not down-regulation when encoding pictures into long-term memory. Memory & Cognition.

