# Supplementary material for: Unintentional forgetting is beyond cognitive control
Source: Cogn Res Princ Implic. 2019 Jul 16;4:25. doi: 10.1186/s41235-019-0180-5 (PMC6635537; doi:10.1186/s41235-019-0180-5)
Supplement: Supplementary file 1 — Experiment 1 post-video quiz. (DOCX 599 kb) [file 41235_2019_180_MOESM1_ESM.docx]

**Additional file 1**

**Experiment 1 Post-video Quiz**

Subject Number:_____________ Date:____________________

1. Which of the following correctly defines recognition-induced forgetting?

a. Recognizing an object from a particular category causes the forgetting of objects in that same category that were not also pulled out of memory.

b. Recognizing a category consequently causes the forgetting of other categories.

c. Recognizing baseline objects induces the forgetting of related objects within a category.

d. Recognizing related objects induces the forgetting of baseline objects within a category.

e. Recognizing an object from a specific category consequently causes the forgetting of objects in a different category.

**Please use this image to answer questions 2-4.**

the forgetting of which type of stimuli?

a. The objects that are presented in the first and last phases of the experiment and whose category is not represented in the second phase

b. New objects presented in the test phase

c. The objects that are shown in every phase of the experiment

d. The objects that are presented in the first and last phases of the experiment and whose category is represented in the second phase

e. Objects that are only presented in the second phase of the experiment

COGNITIVE CONTROL OF FORGETTING 27

7. Accessing which of the objects below from memory would cause the forgetting of a black nylon watch?

a. b. c.
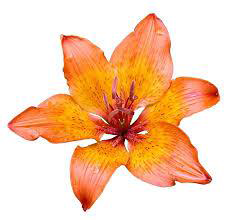


d. e.

8. Which of the following real-life scenarios best demonstrates recognition-induced forgetting?

a. Recognizing an orange flower causes you to forget the orange cat that you saw in the garden.

b. Recognizing a green mug induces the forgetting of a green plate.

c. Recognizing your friend’s black backpack causes you to forget your roommate’s gray backpack.

d. Seeing a red motorcycle in the parking lot causes you to incorrectly identify your friend’s yellow car in a driveway.

e. Seeing a shoe causes you to remember you need to go shoe shopping.

Score_____________________

***********************The below was not provided to subjects. ***********************

*Answer Key*

***1.*** *a*

***2.*** *b*

***3.*** *d*

***4.*** *c*

***5.*** *c*

***6.*** *d*

***7.*** *a*

***8.*** *c*
