# Supplementary material for: Unintentional forgetting is beyond cognitive control
Source: Cogn Res Princ Implic. 2019 Jul 16;4:25. doi: 10.1186/s41235-019-0180-5 (PMC6635537; doi:10.1186/s41235-019-0180-5)
Supplement: Supplementary file 2 — Experiment 1 post-experiment survey. (DOCX 17 kb) [file 41235_2019_180_MOESM2_ESM.docx]

**Additional file 2**

**Experiment 1 Post-experiment Survey**

Subject Number:_______________ Date:____________________

1. On a scale of 1-10, with 1 being the least confident and 10 being the most confident, how confident are you that you were able to overcome the RIF effect? Please circle the number below that best corresponds to your confidence level.

**Least Confident**______________________________________**Most Confident**

1 2 3 4 5 6 7 8 9 10

2. On a scale of 1-10, with 1 indicating the least amount of effort and 10 indicating the most amount of effort, how hard did you try to resist the RIF effect? Please circle the number below that best corresponds to your effort level.

**Least Effort**_____________________________________________**Most Effort**

1 2 3 4 5 6 7 8 9 10

3. In this experiment, you were instructed to try to resist the forgetting effect that occurs within RIF (e.g., forgetting the green apple when shown a red one). What strategy, if any, did you use to accomplish this task? (For example, a strategy could have been focusing on remembering specific categories of images, or it could have been relating the images to your own day to day experiences or memories.) Please explain below in as much detail as you can provide.
