# Supplementary material for: Unintentional forgetting is beyond cognitive control
Source: Cogn Res Princ Implic. 2019 Jul 16;4:25. doi: 10.1186/s41235-019-0180-5 (PMC6635537; doi:10.1186/s41235-019-0180-5)
Supplement: Supplementary file 3 — Individual subject data. (DOCX 13 kb) [file 41235_2019_180_MOESM3_ESM.docx]

**Additional file 3**

**Individual subject data**

**Experiment 1**

In Experiment 1, 14 subjects showed no difference between baseline and related or had better memory for related objects relative to baseline. However, there were no reliable differences on the post-video quiz (**Appendix A**), scored out of 8, between these 14 subjects (6.86/8) and the remaining 24 subjects (6.54, *t*(36)=.639, *p*=.527, JZSNULL=2.64). The 14 subjects were no more confident in their performance after the experiment (6.14/10) than the remaining 24 subjects (6.46, *t*(36)=.705, *p*=.485, JZSNULL=2.54, **Appendix B**, Question #1), nor did they report a higher degree of effort (8.21/10) than the remaining subjects (8.46/10, *t*(36)=.469, *p*=.642, Question #2). Self-reported strategies did not systemically differ between subjects who did and did not show significant recognition-induced forgetting.

**Experiment 2**

In Experiment 2, eleven subjects either showed no difference between baseline and related or had better memory for related objects relative to baseline. The similarity between the proportion of subjects susceptible to forgetting between an informed group (Experiment 1) and a naïve group (Experiment 2) suggests that subjects in Experiment 1 were just as susceptible to forgetting as naïve subjects. Indeed, a pre-planned between-subjects t-test between forgetting (baseline – related) for Experiments 1 (.10) and 2 (.10) shows an insignificant difference, *t*(75)=.099, *p*=.921, JZSNULL=4.22).

Responses to the post-experiment survey (**Appendix D**) confirmed that subject’s strategies were indicative of being naïve to the purpose of the experiment. The subject’s self-reported effort (Question #5) did not differ between the 11 subjects who did not show significant forgetting (8.45) and the remaining subjects (8.61, *t*(37)=.387, *p*=.701, JZSNULL=2.80).

**Experiment 3**

In Experiment 3, one expert subject showed no difference between memory for baseline and related objects.
