# Supplementary material for: Unintentional forgetting is beyond cognitive control
Source: Cogn Res Princ Implic. 2019 Jul 16;4:25. doi: 10.1186/s41235-019-0180-5 (PMC6635537; doi:10.1186/s41235-019-0180-5)
Supplement: Supplementary file 4 — Experiment 2 post-experiment survey. (DOCX 13 kb) [file 41235_2019_180_MOESM4_ESM.docx]

**Additional file 4**

**Experiment 2 Post-experiment Survey**

Subject Number:__________________ Date:____________________

1. What did you think this experiment was about?

2. Based on your answer to question 1, how well do you think you did in this experiment?

**Least Confident**__________________________________________**Most Confident**

1 2 3 4 5 6 7 8 9 10

3. What strategy did you use, if any, in this experiment?

4. How much effort did you put into your strategy?

**Least Effort**_____________________________________________**Most Effort**

1 2 3 4 5 6 7 8 9 10

5. How much effort did you put into this experiment?

**Least Effort**_____________________________________________**Most Effort**

1 2 3 4 5 6 7 8 9 10
